# The role of depressive symptoms in the interplay between aging and temporal processing

**DOI:** 10.1038/s41598-023-38500-3

**Published:** 2023-07-14

**Authors:** Giulia Buzi, Francis Eustache, Arnaud D’Argembeau, Thomas Hinault

**Affiliations:** 1 Inserm, U1077, EPHE, UNICAEN, Normandie Université, PSL Université Paris, CHU de Caen, GIP Cyceron, Neuropsychologie et Imagerie de la Mémoire Humaine (NIMH), 2, Rue des Rochambelles, 14000 Caen, France; 2 Department of Psychology, Psychology and Neuroscience of Cognition Research Unit, University of Liège, Place des Orateurs 1 (B33), 4000 Liège, Belgium

**Keywords:** Human behaviour, Psychology

## Abstract

Temporal processing, the ability to mentally represent and process the dynamical unfolding of events over time, is a fundamental feature of cognition that evolves with advancing age. Aging has indeed been associated with slower and more variable performance in timing tasks. However, the role of depressive symptoms in age-related changes in temporal processing remains to be investigated. Therefore, the present work aims to shed light on the link between temporal processing and depressive symptoms, which are frequent with advancing age. We relied on the multicentric “Blursday Project” database, providing measures of temporal processing together with questionnaires investigating psychological wellbeing. Results reveal that aging influences several timing abilities, from the reproduction of short time intervals to verbal estimations of longer temporal distances. Furthermore, the slowing down of felt passage of time regarding the last few days with age was fully mediated by the intensity of depressive symptoms. Overall, these findings suggest that depressive symptoms may play a pivotal role in age-related temporal processing changes.

## Introduction

Aging is associated with several cognitive changes involving memory, attention, and executive control^1^. A slowing down of the speed of information processing, as well as decline in working memory and episodic memory, are among the most frequently reported features of cognitive aging^2^. However, age-related cognitive changes are highly heterogenous, as several factors interact in individual cognitive trajectories^3^. As an example, the presence of even subclinical forms of depression (that is, that do not meet diagnostic criteria for clinical depression disorder) have been associated with higher risk of cognitive decline in cognitively unimpaired older adults^4,5^.

Temporal processing, the processing and maintenance in memory of the dynamical unfolding of events over time, are essential to the functioning of humans and animals^6^. The precision of temporal representations has important implications for cognitive functioning, well-being, and daily life activities^7^: However, the sense of time in aging could be affected by several factors, such as task load^8^, mental states^9^, and levels of arousal induced by the context^10,11^. Detecting such factors could contribute to further our understanding of the heterogeneity of age-related trajectories of cognitive decline. Here, we investigated how the perception and representation of time evolve throughout the lifespan across several temporal processes and time scales, while considering differences across individuals, such as the presence of depressive symptoms.

A wealth of experimental research has investigated temporal processing, greatly advancing our understanding of psychological and neural mechanisms underlying the perception and encoding of temporal information, yet a standard conceptual framework is still lacking^12^. Here, we will use the term “Temporal Processing” to cover different temporal scales and four main temporal aspects^13^: duration processing, temporal order and simultaneity, feeling of the passage of time, mental time travel^14^. Duration processing is typically assessed on short time scales (i.e., milliseconds, seconds), and can involve productions of specific durations (e.g., pressing a button for two seconds), reproductions of stimuli durations, or comparisons between stimuli durations (i.e., determining which stimulus is the shortest/longest). Order and simultaneity can be assessed when participants perform tapping tasks to produce or pursue an initiated rhythm or stay in synchrony with a cued rhythm. The Passage Of Time Judgements (POTj) and Mental Time Travel (MTT) (i.e., the ability to relive past experience and to imagine future events) can notably be quantified using questionnaires^15^ assessing the subjective temporal distance at larger time scales (i.e., weeks, months, years) for both past and future events^16^. These different paradigms require the efficient functioning of several cognitive functions, which are supported by distinct neural underpinnings^17,18^. For instance, performance in duration processing tasks has been associated with attention, processing speed, and working memory abilities^19^. At the neural level, duration processing has been associated with activations of frontal and parietal cortices including the insular, superior/inferior frontal gyri, the superior temporal gyrus, and the striatum^20^. M/EEG studies reported an association between sustained synchronized activity between frontal and parietal regions and the maintenance of temporal representations and the detection of deviations^21^. Regarding the feeling of passage of time, previous work reported associations with episodic memory^22^ and consciousness^23^. Similarly, the mental time travel to the past (episodic autobiographical memory) and to the future (episodic future thinking are thought to rely on a partially shared set of brain regions including medial temporal and frontal lobes, posterior cingulate and retrosplenial cortex, and lateral parietal and temporal areas^24^. From a neural point of view, sustained brain oscillations and ramping brain activity over time have been interpreted as reflecting accumulation of information over time^25^. Ramping activity over time has been associated with activity of the supplementary motor area, and the posterior insula^26^.

Gradual changes of temporal processing with advancing age have been previously reported^27–29^, with older individuals showing reduced precision across temporal processing tasks^17,30^. Moreover, older individuals produce longer inter-tap intervals (ITI) than younger adults when required to tap at their preferred rate for 30 s (i.e., spontaneous motor tapping task (SMT), resulting in a slowing down of the motor tempo^31,32^. However, contrasted findings have been reported regarding the evolution of the subjective feeling of the passage of time with advancing age^33^. As an example, some studies investigating the passage of time found no or little evidence for changes in the rate of the feeling of time passing with age, over different periods (i.e., weeks, months, years, decades)^34,35^, while others reported a slower passage of time feeling in people older than 75 years old^36^.

Although there is still a lack of consensus in the previous literature regarding the reason why time may pass differently with age, the effect of negative emotions on temporal judgements is well known^37^ notably in the older population and could thus play a role in this process. For instance, Droit-Volet^7^ used an Experience Sampling Methodology (ESM) and found that both young and older participants reported a slowing down of the passage of present time when they felt sad. While this relationship should be further investigated, this suggests a pivotal role of affective states on the felt passage of time^38^.

It has been suggested that altered temporal estimations in older adults may either be related to an attention deficit linked to an insufficient focus of attention on temporal information^39^, or to noisy duration representations related to reduced working memory capacities^40^. Nonetheless, aging effects and the interplay of individual characteristics on the mechanisms supporting temporal processing changes remain to be clarified, with a need to further consider the heterogeneity of changes across timing abilities and temporal scales with advancing age. One major factor contributing to cognitive decline in the aging population is the presence of depressive symptoms^41,42^. Moreover, recent behavioral meta-analyses^43,44^ revealed alterations in the encoding and representation of short and longer durations in individuals with depressive symptoms relative to controls. Depressed individuals indeed tend to overproduce short durations and underproduce long duration intervals (i.e., from 400 ms to 30 s)^45^. Detecting such changes of temporal processing, notably in the aged population, could be insightful to uncover the presence of subclinical depressive syndromes, thereby contributing to the prevention of cognitive decline^5^.

To shed light on the interplay between aging effects and depressive symptoms on the evolution of temporal processing, we relied on the Blursday database^46^, which includes repeated temporal processing measures together with a variety of questionnaires investigating the psychological well-being of 2840 participants in over nine countries, ranging from 18 to 80 years. This project documents the effects of social isolation of lockdown measures on the subjective experience of time as Covid lockdown measures significantly affected on temporal processing^47,48^. However, as our goal was to investigate the effect of depressive symptoms on the relationship between temporal processing and aging as independently of these events as possible, analyses were restricted to the control session (i.e., outside lockdown) of this database. To our knowledge, no study to date has integrated several temporal processing tasks across different time scales to assess the effects of aging, and the contributing role of depressive symptoms. In the light of previous findings on aging and time estimation, we could anticipate a decrease of precision and a larger variability on tasks tied to duration processing with advancing age^17^. Moreover, we also expected changes in temporal aspects of cognition with aging, to be driven by the presence and intensity of depressive symptoms. Since temporal processing and cognitive functions are intertwined^49^, disentangling the link between age-related temporal processing changes from concurrent factors could contribute to explaining the inter-individual differences observed between different trajectories of cognitive decline across individuals.

### Methods

To investigate the relationships between temporal processing and depressive symptoms with aging, we relied on the Blursday Project’s repository whose data is freely accessible under the Gorilla Open Materials Attribution Non-Commercial Research-Only licensing: https://app.gorilla.sc/openmaterials/278377. An informed consent was obtained from all participants.

### Sample

Data were extracted from a sample of 388 participants from the control session of the study (naive participants tested outside the most severe measures of lockdowns). However, 17 participants were excluded due to a lack of basic demographic information (i.e., age and gender). After removing outliers in the age variable by excluding values above 2.5 SD of the mean (n = 2), we obtained a sample of 369 participants aged between 18 and 69 years (Females = 261, M_Age_ ± SD_Age_: 31.9 ± 13.9; Males = 108, M_Age_ ± SD_Age_: 35.7 ± 14), living in France (n = 172; Females: 130, Males: 42; M_Age_ ± SD_Age_:31.5 ± 12.4), Germany (n = 75; Females: 62, Males: 13; M_Age_ ± SD_Age_: 24 ± 5), Italy (n = 47; Females: 33, Males: 14; M_Age_ ± SD_Age_: 32.6 ± 15.8), and Japan (n = 75; Females: 36, Males: 39; M_Age_ ± SD_Age_: 45.6 ± 13.6).

**Figure 1:**
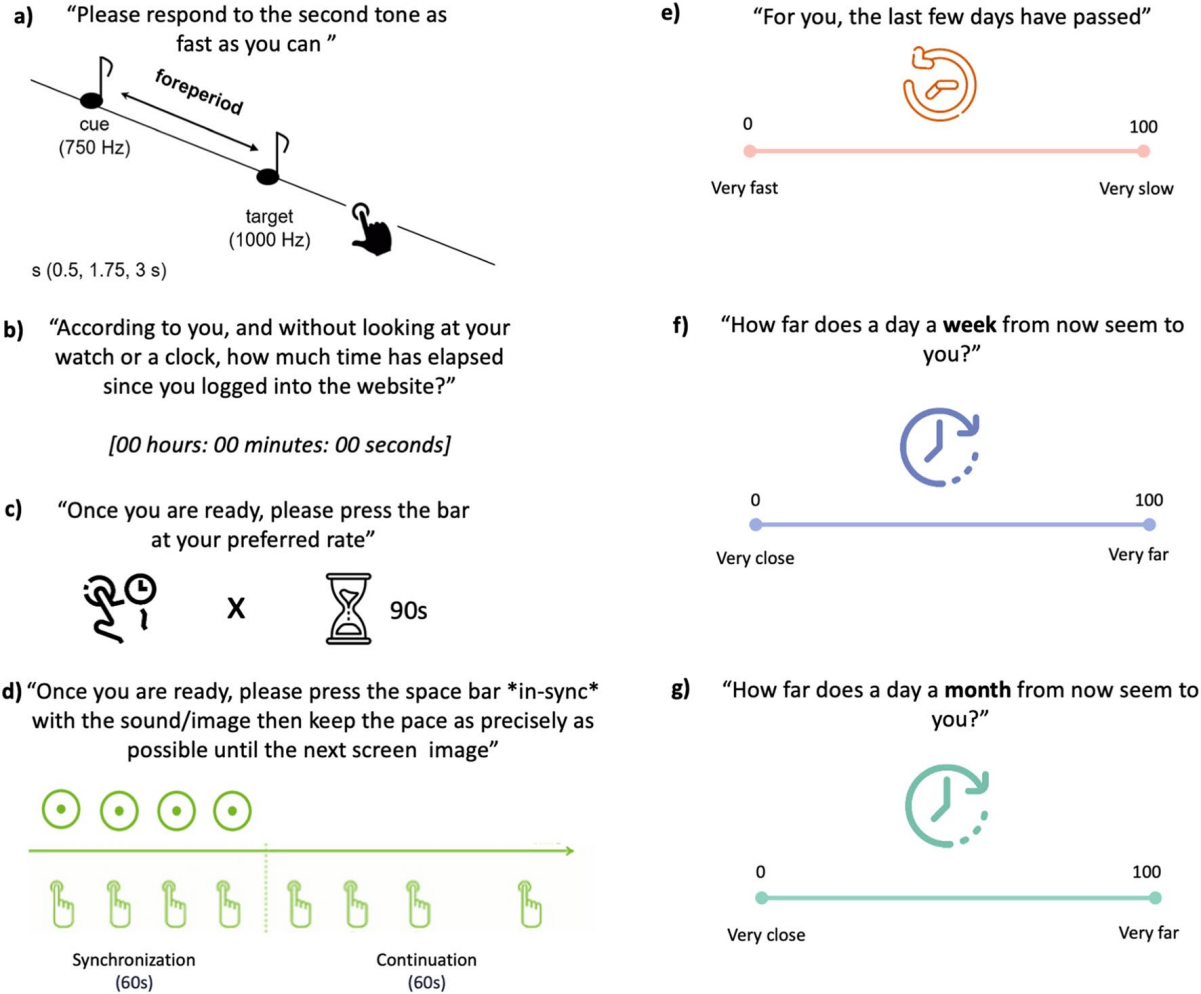
Graphical representation of behavioral tasks and questionnaires adapted from “The Blursday Project”. (**a**) Foreperiod Implicit Timing Task. (**b**) Retrospective Duration. (**c**) Spontaneous Tapping. (**d**) Synchronization-Continuation Task (**e**) Passage of Time. (**f**) Subjective Temporal Distance of the next week. (**g**) Subjective Temporal Distance of the next month.

As the Control Session of the Blursday Database from which the data have been extracted (n = 369) contained a great number of participants between 20 and 30 years old (n = 218) and between 30 and 40 years old (n = 60), we extracted a subsample by the “rand()” function in Excel from each age subgroup, but respecting the proportion of the world origin and sex (n = 148, M_age_ = 45.9 ± 14.420, M = 70, F = 78), to compute the robust One-way ANOVAs, divided in age groups of equal sizes (χ^2^_4_ = 1.65, *p* = 0.799), composed as follows: n (20–30) = 28; n (30–40) = 27; n (40–50) = 28, n (50–60) = 35; n (60–80) = 30. We thus conducted additional analyses on a sub-sample balanced based on these factors, as probed by the Chi-square test for proportions with continuity correction applied (χ^2^_4_ = 1.65, *p* = 0.799), to control for multiple comparisons. The contingency tables showing the frequencies of country and sex are showed in Supplementary Tables S1A and S1B. A graphical representation of the cultural composition of the sample is provided in Fig. 1.

Unfortunately, not every subject participating to the Control Session of the Blursday Project completed all tasks and questionnaires, resulting in a different number of participants for each of the tasks (shown in Table 1): Foreperiod Implicit Timing (n = 124), Retrospective duration (n = 73), Spontaneous Tapping (n = 130), Synchronization-Continuation (n = 145), Passage of Time (n = 135), Subjective Temporal Distance (Weeks) (n = 122), Subjective Temporal Distance (Months)(n = 122). However, all the tasks were analyzed separately using robust One-Way ANOVAs, and missing values were automatically excluded for each one of the mediations analyses.

**Table 1:** Descriptive statistics of the culture and gender balanced sub-group, divided in 5 age groups. Mean, standard error of the mean (SE), standard deviation (SD) of age and temporal processing tasks performances in z-scores, for each age group, with the p value of the significance to Shapiro–Wilk test for normality.

	n	Age Group	n	Missing	Mean	SE of the Mean	SD	Shapiro–Wilk	*P*-value of Shapiro–Wilk	Min	Max
Age	148	20–30	28	0	25.296	0.472	2.498	0.966	0.486	19.900	29.800
		30–40	27	0	34.589	0.579	3.009	0.927	0.057	30.100	39.800
		40–50	28	0	45.568	0.599	3.168	0.914	0.024	40.500	49.900
		50–60	35	0	55.400	0.546	3.228	0.877	0.001	50.100	59.600
		60–80	30	0	64.553	0.827	4.531	0.780	< .001	60.200	80.200
HADS-D (z)	148	20–30	28	0	0.289	0.207	1.093	0.943	0.130	− 1.307	2.579
		30–40	27	0	0.735	0.178	0.924	0.980	0.862	− 1.307	2.822
		40–50	28	0	0.202	0.198	1.050	0.947	0.165	− 1.307	2.579
		50–60	35	0	− 0.065	0.186	1.100	0.930	0.028	− 1.550	2.093
		60–80	30	0	0.101	0.192	1.051	0.951	0.185	− 1.550	2.579
HADS-A (z)	148	20–30	28	0	− 0.068	0.177	0.939	0.961	0.360	− 1.517	1.941
		30–40	27	0	− 0.117	0.193	1.005	0.968	0.540	− 1.747	1.941
		40–50	28	0	− 0.372	0.183	0.967	0.949	0.182	− 1.747	1.711
		50–60	35	0	− 0.404	0.153	0.908	0.946	0.084	− 1.747	1.480
		60–80	30	0	− 0.549	0.207	1.135	0.844	< .001	− 1.747	2.402
Confinement Index (z)	88	20–30	27	1	0.269	0.136	0.705	0.971	0.634	− 1.060	1.940
		30–40	27	0	0.277	0.251	1.304	0.942	0.133	− 1.721	2.703
		40–50	28	0	0.038	0.159	0.843	0.967	0.506	− 1.721	2.296
		50–60	35	0	− 0.098	0.154	0.912	0.922	0.017	− 1.467	2.143
		60–80	30	0	− 0.128	0.186	1.020	0.956	0.247	− 1.721	2.296
Foreperiod implicit timing (z)	124	20–30	23	5	0.253	0.211	1.011	0.961	0.478	− 1.230	2.395
		30–40	23	4	− 0.040	0.170	0.815	0.953	0.339	− 1.268	1.474
		40–50	23	5	0.196	0.233	1.119	0.869	0.006	− 1.164	3.652
		50–60	29	6	0.019	0.181	0.975	0.751	< .001	− 0.908	2.930
		60–80	26	4	− 0.028	0.192	0.980	0.808	< .001	− 1.164	3.652
Retrospective duration (z)	73	20–30	13	15	0.027	0.152	0.549	0.802	0.007	− 0.517	1.564
		30–40	14	13	− 0.221	0.083	0.310	0.793	0.004	− 0.595	0.700
		40–50	15	13	− 0.165	0.063	0.245	0.902	0.101	− 0.469	0.346
		50–60	18	17	0.095	0.148	0.627	0.830	0.004	− 0.517	1.900
		60–80	13	17	0.179	0.191	0.688	0.708	< .001	− 0.327	1.900
Spontaneous tapping (z)	130	20–30	28	0	− 0.144	0.015	0.079	0.807	< .001	− 0.227	0.057
		30–40	25	2	0.638	0.594	2.968	0.313	< .001	− 0.226	14.147
		40–50	25	3	− 0.126	0.018	0.089	0.898	0.017	− 0.234	0.090
		50–60	31	4	− 0.067	0.091	0.506	0.263	< .001	− 0.221	2.628
		60–80	21	9	− 0.107	0.057	0.260	0.414	< .001	− 0.239	1.000
Synchronization-Continuation (z)	145	20–30	28	0	− 0.214	0.130	0.688	0.906	0.016	− 1.101	1.135
		30–40	27	0	0.265	0.167	0.866	0.932	0.077	− 0.986	2.326
		40–50	28	0	− 0.295	0.200	1.060	0.758	< .001	− 1.425	2.944
		50–60	35	0	− 0.021	0.159	0.939	0.970	0.452	− 1.654	2.695
		60–80	27	3	0.578	0.309	1.608	0.919	0.037	− 1.800	3.108
Passage of time (z)	135	20–30	24	4	0.011	0.202	0.990	0.955	0.351	− 2.295	1.379
		30–40	22	5	− 0.195	0.236	1.105	0.946	0.259	− 2.411	1.379
		40–50	27	1	− 0.122	0.174	0.904	0.945	0.164	− 2.025	1.379
		50–60	34	1	0.126	0.170	0.993	0.869	< .001	− 2.295	1.379
		60–80	28	2	0.049	0.185	0.981	0.921	0.037	− 1.715	1.379
Subjective temporal distance (w) (z)	122	20–30	28	0	0.241	0.176	0.932	0.965	0.458	− 1.098	2.304
		30–40	24	3	− 0.225	0.160	0.785	0.815	< .001	− 1.098	1.349
		40–50	23	5	− 0.214	0.173	0.829	0.865	0.005	− 1.098	1.807
		50–60	29	6	− 0.240	0.157	0.847	0.848	< .001	− 1.098	2.075
		60–80	18	12	− 0.268	0.175	0.741	0.896	0.049	− 1.098	1.463
Subjective temporal distance (m) (z)	122	20–30	28	0	− 0.051	0.206	1.089	0.860	0.001	− 1.378	1.449
		30–40	24	3	− 0.154	0.196	0.960	0.893	0.015	− 1.378	1.232
		40–50	23	5	− 0.211	0.170	0.815	0.905	0.032	− 1.378	1.698
		50–60	29	6	− 0.133	0.156	0.841	0.936	0.080	− 1.378	1.698
		60–80	18	12	− 0.278	0.188	0.797	0.956	0.527	− 1.378	1.636

### Task description

The enrolled subjects to the Blursday Project, first completed the questionnaires administered in a random order across participants, most of which they had to take once per session. After the questionnaires, they accomplished a series of diverse pseudo-randomly ordered (latin-square design) behavioral tasks. Individual scores on The Hospital Anxiety and Depression Scale^50^ (HADS-A and D) were considered to quantify self-perceived depressive and anxiety symptoms in the last weeks. In addition, we extracted information about previous lockdown stringency^51^: A composite score of nine governmental response indicators including school closures, workplace closures and travel bans, was rescaled to values ranging from 0 to 100, with 100 being the strictest stringency on the restrictions index.

The following temporal processing tasks were considered (Fig. 1): the Foreperiod Implicit Timing task^52^, an auditory reaction time task in which participants were asked to respond as fast as possible to a series of target tones, which were preceded by cue tones. The Foreperiod (i.e., time interval between the cue and target tone (2 runs) was one of three durations (500 ms, 1750 ms and 3000 ms), presented as either fixed (3 trials) or variable (3 trials) throughout one block. In total, participants realized 6 blocks of 9 trials each. To quantify the imprecision and to avoid confounding slowing effects on sensorimotor response due to physiological aging, we computed the standard deviation of the reaction time to the fixed Foreperiod condition. Then, the retrospective duration task considers the individual estimation of elapsed time to perform the tests on the Blursday platform, spanning from several minutes to hours. The estimates were transformed to seconds to allow for comparisons. In the spontaneous finger tapping task (2 runs) participants had to press the spacebar at their preferred rate for 90 s^32^^.^ To assess the internal production of time intervals, we considered the total time divided by the number of taps, thus obtaining a mean of the individual intertap interval. In the Synchronization-Continuation task^53,54^ (2 runs) participants were asked to synchronize themselves with an audio/visual stimulus presented on a screen at a rate of 1.3 Hz (78 Beats per minute) for 60 s. The standard deviation of the cue’s fixed interval (1 s) during the synchronization phase and the intertap interval (ITI) during continuation was considered as an index of asynchrony^55^.

The “passage of time” judgment about how fast or slow individuals’ passage of time appeared in the last few days, was expressed by participants on a Visual Analog Scale (VAS) ranging from 0 (“very slow”) to 100 (“very fast”). Finally, the subjective temporal distance from the next week and month was assessed by asking participants, “How far does a day a week from now seem to you?” and “How far does a day a month from now seem to you?” on a VAS from 0 to 100, where 0 corresponded to “very close” and 100 to “very far”.

An overview of the runs and stimuli used are described in Supplementary Fig. 2 and Table 1 of the original paper of the Blursday Project’s Database (Chaumon et al.^46^, Supplementary Information). Both provide a comprehensive description of the content of each session.

### Statistical analyses

All analyses were performed with JASP (v. 0.16.4) and R (cran.r-project.org/). After conducting descriptive statistical analyses, the data’s normality was assessed using the Shapiro–Wilk test. Variables were z-score transformed prior to statistical analyses, to obtain a unique scale, therefore accounting for the different response type and time scales (spanning from milliseconds to months) between the analyzed tasks. Because normality assumptions were violated for several variables, we used robust statistical methods^56^ to assess differences between the 5 age-groups on temporal tasks performances. More specifically, we computed seven One-Way Robust Analyses of the Variance (Robust ANOVAs) using the functions “t1waybt” and “mcppb20” (for post-hoc comparisons), implemented in the packages “robustbase” and “WRS2” in R. We applied corrections for outliers by trimming the 20% of the upper and lower bounds of the data and we dealt with the violations of the assumptions for parametric tests by bootstrapping method^57^ with 2000 repetitions^58^*.*

To control for possible cultural variations in the expression of sadness feelings, we computed a parametric 5*2 parametric ANOVA between Age Groups and Region of the world on the HADS-D mean score (Supplementary Table S2A) as normality test was not significant (Shapiro–Wilk’s = 0.986, *p* = 0.142) as well as the Levene’s test for homogeneity of the variances (F_(9,138)_ = 1.33, *p* = 0.255). Post-hoc analyses were corrected for multiple comparisons by applying the Bonferroni correction^59^ and shown in the Supplementary Table S2B.

Then, to investigate whether the intensity of depressive symptoms could mediate the relationship between the evolution of temporal productions and time estimations abilities across the lifespan we estimated the total effect (Path c) of aging (X = continuous predictor) on temporal processing (Y) and the indirect effects of the depressive symptoms (Path ab, mediator = HADS-D) for each one of the tasks considered: Foreperiod Implicit Timing (n = 299), Retrospective Duration (n = 175), Spontaneous Tapping (n = 321), Synchronization-Continuation (n = 364), Passage of Time (n = 306), Subjective Temporal Distance (Weeks) (n = 290), Subjective Temporal Distance Months (n = 290). As our primary purpose was to study the role of depression, we controlled for anxiety and confinement index as continuous background confounding variables.

As the whole sample (n = 369) did not respect the proportions of biological sex (F = 0.707; M = 0.293, χ^2^ = 62.8, *p* < 0.001) nor for the origin of the world [Europe (n = 294), Asia (n = 75), χ^2^ = 129, *p* < 0.001)] we conducted the mediation analyses on the balanced subsample of participants while controlling proportions of both biological sex and region of the world. Coefficients were completely standardized, and direct and indirect effects were computed with Bias-Corrected (BC) bootstrap, 95% confidence intervals based on 5000 bootstrap samples.

## Results

Statistical descriptive analyses including the mean, standard deviation, and p values of the Shapiro–Wilk’s Test for normality on the temporal tasks and to the HADS scores, are shown in Table 1**.** Results of ANOVAs investigating cultural differences on HADS-D scores between age groups are shown in Supplementary Tables S2A–2C.

### Temporal processing abilities across different age groups

**Figure 2:**
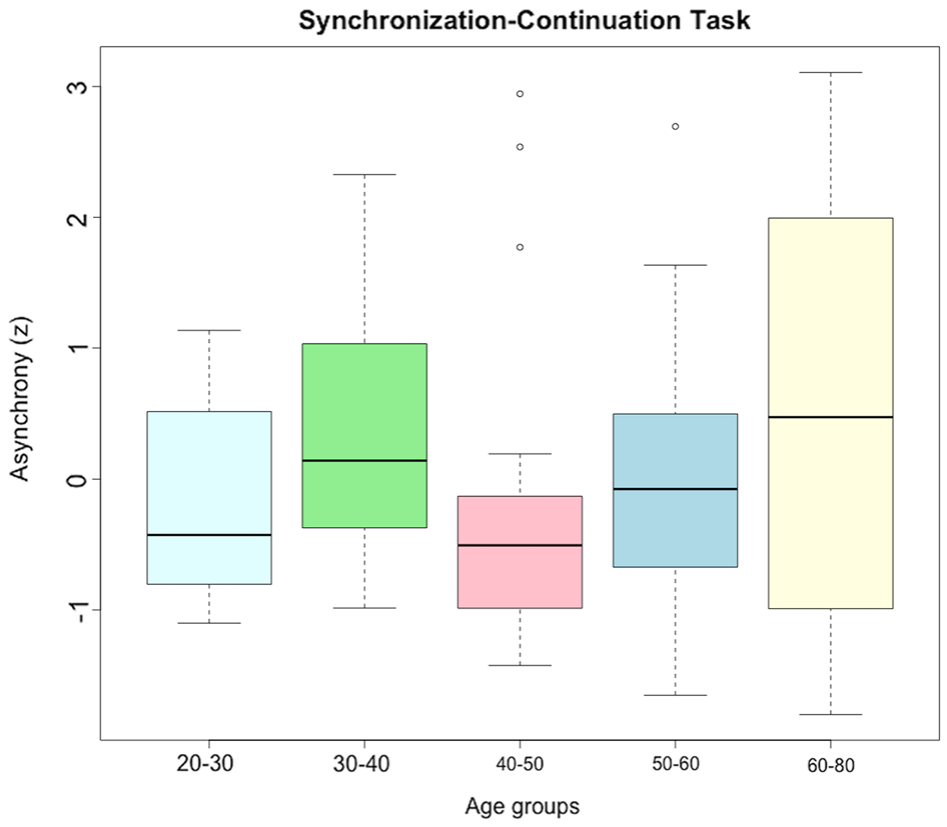
Boxplots showing the Asynchrony (y-axis) at the Synchronization-Continuation task (z-scores) for each age group (x-axis).

The One-Way robust ANOVA probing the effect of age (5 age groups) on the performances to the 7 timing tasks revealed a statistically significant difference in the Asynchrony in performing the Synchronization-Continuation Task (F_test_ = 3.110, *p* = 0.05, effect size = 0.382, Table 2). Bonferroni’s Test for multiple comparisons found that the asynchrony was significantly greater in the oldest group (60–80) compared to the adult one (40–50) ($\widehat{\psi }$= − 1.017, 95% CI [− 2.233; 0.166], *p* = 0.017) and detected a significant difference between the 30–40 compared to the 40–50 years old group ($\widehat{\psi }$ = 0.716, 95% CI [0.001; 1.429], *p* = 0.004) (Fig. 2; Supplementary Table S3).

**Table 2:** Robust One-way ANOVA between age groups for all timing tasks analyzed.

	F_test_	*p*-value	R^2^	Effect size
Foreperiod implicit timing (z)	0.795	0.524	0.034	0.12
Retrospective duration (z)	1.775	0.154	0.110	0.33
Spontaneous tapping task (z)	0.618	0.671	0.060	0.24
**Synchronization-continuation (z)**	**3.110**	0.05*	0.146	**0.382**
Passage of time (z)	1.1264	0.353	0.044	0.209
Subjective temporal distance (w) (z)	1.496	0.272	0.076	0.276
Subjective temporal distance (m) (z)	0.232	0.909	0.022	0.147

*= *p* < 0.05.

Significant values are in bold.

### GLM mediation analysis

#### Direct effect of aging on temporal processing (c’ path)

**Figure 3:**
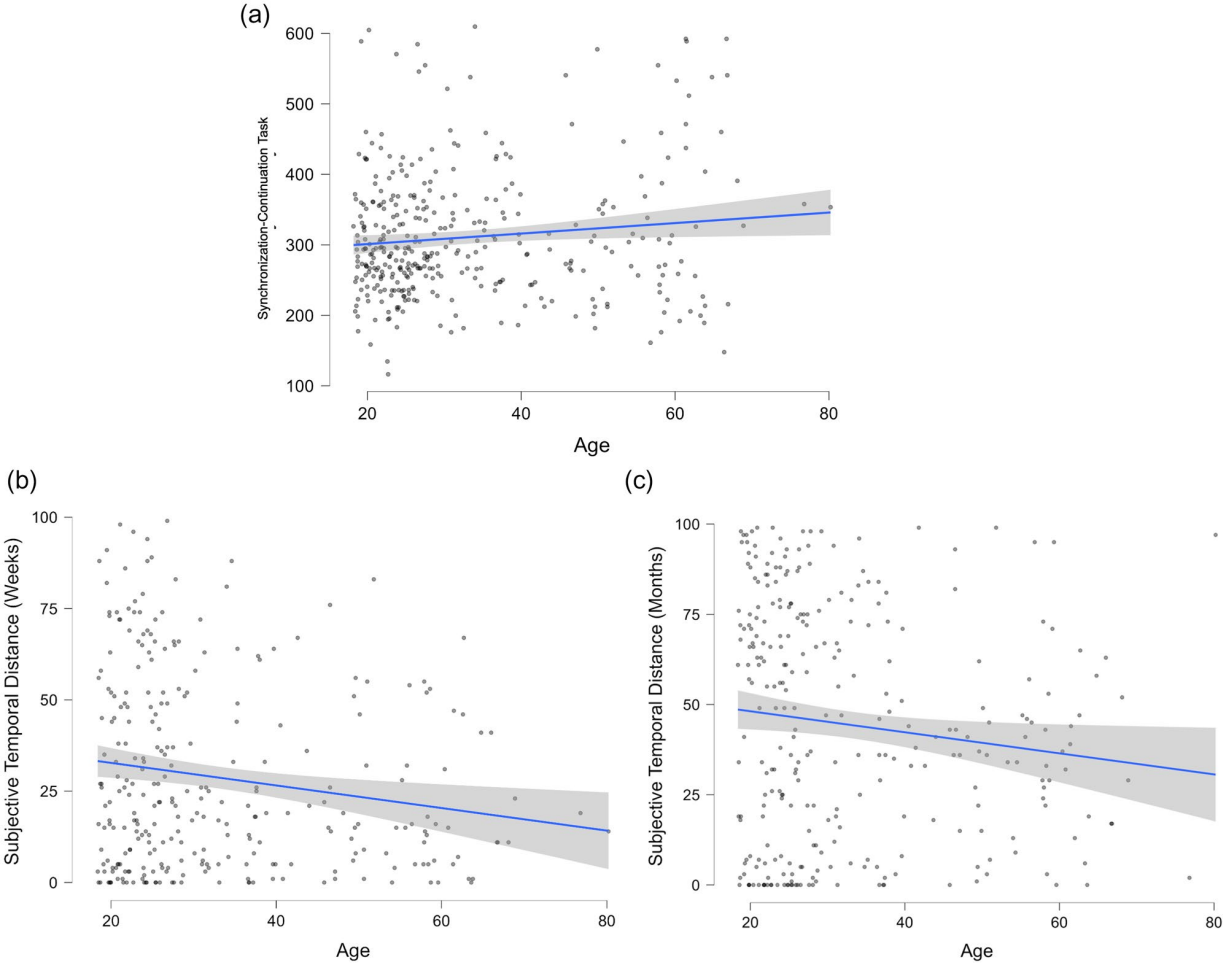
Direct Effects of Age on Temporal Processing. Dots represent participants’ data. For illustrative purposes, variables are not scaled in the plot. Panel (**a**) Linear Regression of the effect of age (x-axis) on Asynchrony (y-axis) at the Synchronization-Continuation task. Panel (**b**) Linear Regression of the effect of age (x-axis) on Subjective Temporal Distance of the next week Panel (**c**) Linear Regression of the effect of age (x-axis) on Subjective Temporal Distance of the next month.

The analysis of the direct effect of age on temporal processing performance highlighted a positive relationship with asynchrony, suggesting that the ability to maintain the pace after observing a cue, decreases with advancing age (β = 0.126, z = 2.043, *p* = 0.041, 95% CI [0.004; 0.223], Fig. 3, panel a). An effect of aging was also observed on the subjective temporal distance of the following week (β = − 0.206, z = − 2.972, *p* = 0.003, 95% CI [− 0.332; − 0.081], Fig. 3, panel b) and month (β = − 0.139, z = − 2.016, *p* = 0.044, 95% [− 0.265; − 0.007], Fig. 3, panel c) which were perceived as shorter by older participants.

#### Direct effect of aging on depressive symptoms (path a)

**Figure 4:**
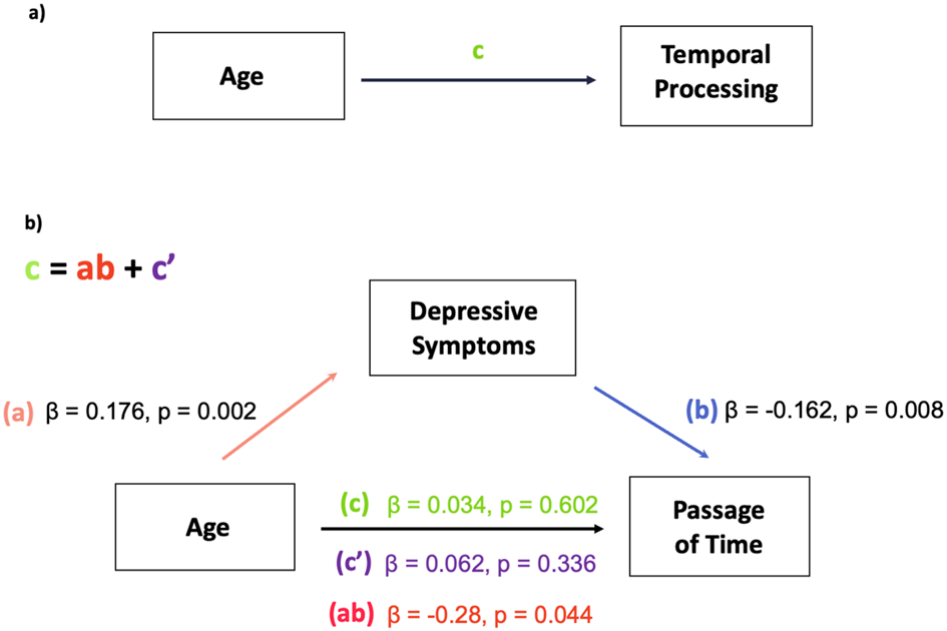
Path plot. Path diagram for (a) the total effect of aging (independent variable) on the performances on each temporal processing task and (b) the standardized coefficients of the indirect effect of aging (X) on the dependent variable through the HADS-D score (mediator variable).

Results of simple regression analyses for each path coefficient, notably highlighted that aging positively affected the HADS-D score (β = 0.176, z = 3.060, p = 0.002, 95% CI [0.070;0.382]). Results of total effects and all path coefficients computed are shown in Supplementary Materials, Tables S4 and S5. The path plot showing these path coefficients is depicted in Fig. 4 (Table 3).

**Table 3:** Direct effects of the age (X) on each one of the temporal tasks (Y). Coefficients are completely standardized, bias-corrected percentile bootstrap confidence intervals, ML estimator. NB: Not all bootstrap samples were successful: CI based on 4999 samples.

	Direct effects								
			n	Estimate	SE	Z-value	*p*	95% Interval	Confidence
								Lower	Upper
Age (z)	→	Foreperiod Implicit Timing (z)	299	0.028	0.068	0.413	0.680	− 0.107	0.158
Age (z)	→	Retrospective Duration (z)	175	− 0.025	0.090	− 0.281	0.778	− 0.261	0.155
Age (z)	→	Spontaneous Tapping Task (z)	321	0.022	0.072	0.299	0.765	− 0.041	0.096
**Age** (z)	** → **	**Synchronization-Continuation** (z)	364	**0.126**	**0.062**	**2.043**	0.041*	− **0.022**	**0.278**
Age (z)	→	Passage of time (z)	306	0.062	0.064	0.963	0.336	− 0.063	0.185
**Age** (z)	** → **	**Subjective temporal distance (w)** (z)	290	− **0.206**	**0.069**	− **2.972**	0.003*	− **0.332**	− **0.081**
**Age** (z)	** → **	**Subjective temporal distance (m)** (z)	290	− **0.139**	**0.069**	− **2.016**	0.044*	− **0.265**	− **0.007**

Significant values are in bold.

#### Investigating the role of depressive symptoms on the relationship between temporal processing and aging (path ab)

**Figure 5:**
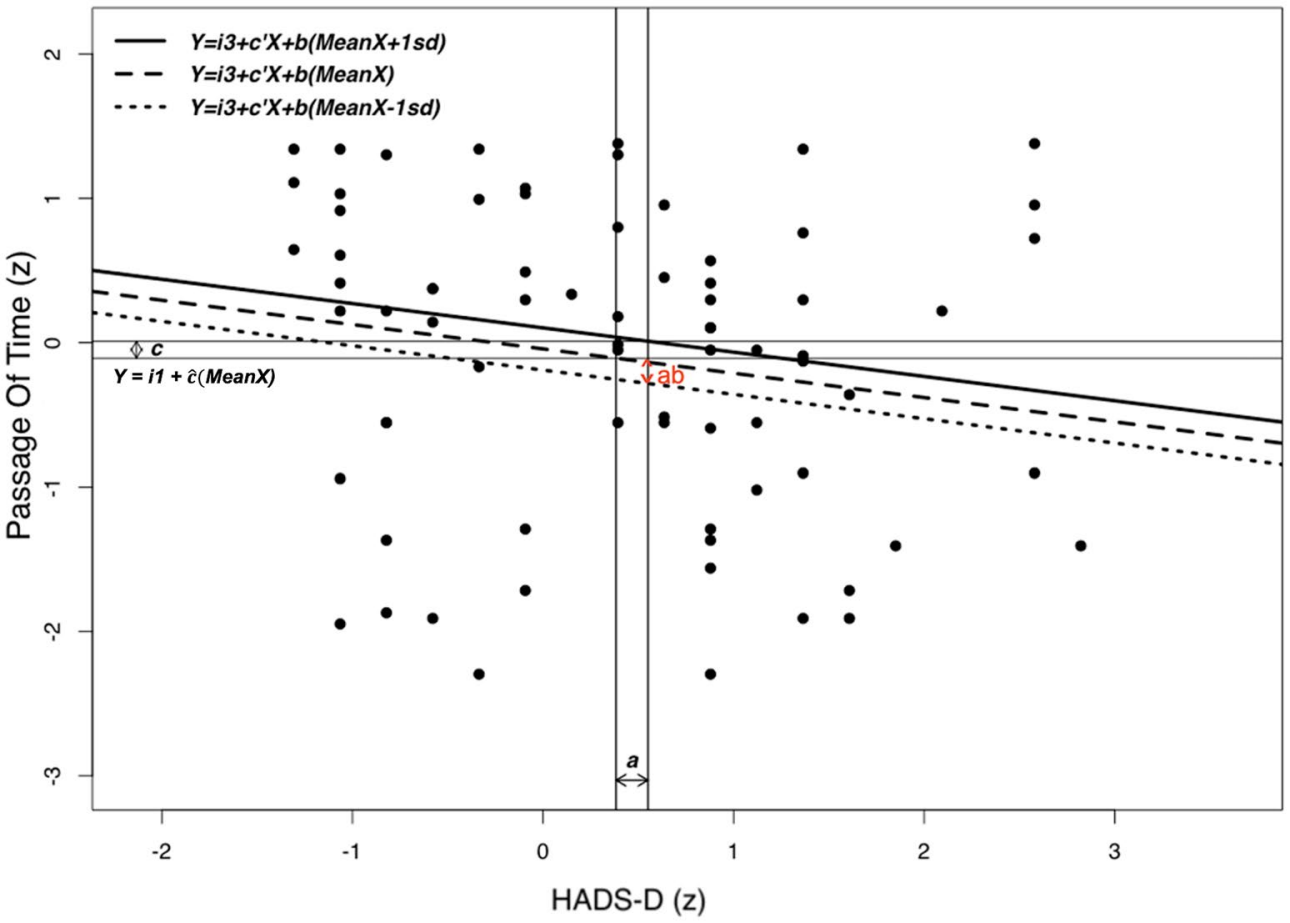
Plot of the mediated effect of the HADS-D on the relationship between Aging and Passage of Time. The distance between the horizontal lines represents the total effect of Aging (X) on Passage Of Time (Y), $\widehat{c}$ and the distance between the two vertical lines is equal to $\widehat{a}$. The slope of each of these lines is equal to b. The distance from the intersection point to the Y = i1 + $\widehat{c}($MeanX) horizontal line is equal to the mediated effect, ab. Note that SD stands for the standard deviation of X. For convenience, hats were not included over the parameter estimates in the plot.

By analyzing the indirect effects computed using the GLM mediation to further elucidate the role of depressive symptoms in the relationship between temporal processing and aging (Table 4), we observed that the felt passage of time was fully mediated by the intensity of depressive symptoms (Fig. 5)(β = − 0.028, z = − 2.011, *p* = 0.044, 95% CI [− 0.068; − 0.008]). Furthermore, we replicated the indirect effect of depression on the slowdown of the passage of time in the subsampled controlled based on gender and culture (n = 148) (β = 0.03, z = 2.131, *p* = 0.03, 95% CI [0.004; 0.223]), Supplementary Tables S6A–6D).

**Table 4:** Indirect effects (path ab) of the z-transformed age (X) on each one of the temporal tasks considered (Y).

	Indirect effects										
					n	Estimate	SE	z-value	*p*	95% Interval	Confidence
										Lower	Upper
Age (z)	→	HADS-D (z)	→	Foreperiod Implicit Timing (z)	299	− 0.021	0.014	− 1.538	0.124	− 0.059	− 0.002
Age (z)	→	HADS-D (z)	→	Retrospective Duration (z)	175	− 0.007	0.014	− 0.511	0.610	− 0.027	0.006
Age (z)	→	HADS-D (z)	→	Spontaneous Tapping Task (z)	321	− 0.014	0.012	− 1.130	0.258	− 0.038	− 0.003
Age (z)	→	HADS− D (z)	→	Synchronization-Continuation (z)	364	0.003	0.010	0.281	0.778	− 0.018	0.026
**Age (z)**	** → **	**HADS-D (z)**	** → **	**Passage of time (z)**	**306**	− **0.028**	**0.014**	− **2.011**	0.044*	− **0.068**	− **0.008**
Age (z)	→	HADS-D (z)	→	Subjective temporal distance (w) (z)	290	− 0.006	0.011	− 0.571	0.568	− 0.033	0.013
Age (z)	→	HADS-D (z)	→	Subjective temporal distance (m) (z)	290	− 0.013	0.012	− 1.150	0.250	− 0.042	0.005

Significant values are in bold. Coefficients are completely standardized, bias-corrected percentile bootstrap confidence intervals, ML estimator. *NB* not all bootstrap samples were successful: CI based on 4999 samples.

## Discussion

The present study aimed at further specifying the effects of age and depressive symptoms on temporal processing. Our primary goal was to identify the aspects of time processing showing the most significant age-related changes. Then, because of its prevalence in the older population^60^ and its impact on cognitive functions^42^ we wanted to elucidate the interplay between aging and depressive symptoms effects on various aspects of temporal processing.

To this end, we relied on data from the large-scale Blursday Project, which provided several temporal processing measures and psychological well-being questionnaires^46^. A general linear model of mediation was carried out to consider the performance of each temporal processing measure, their association with age, and the mediation effect of depressive symptoms’ intensity.

Analyses of the relationship between age and temporal processing revealed age-related effects on several aspects of time processing. Overall, previous findings observed that the timing abilities in the hundreds to milliseconds range relies on attention and working memory processes^19^ which are known to decline with physiological aging^2^. We observed that the precision at the continuation-synchronization task decreased with advancing age. These results have also been confirmed by the analysis of variance investigating between groups differences on the timing tasks, highlighting a significant difference between the adult and the oldest group. The Sensorimotor synchronization (SMS), defined as the ability to synchronize to regularities in an external temporal structure^61^ is crucial not only for producing music or dancing, but also in a wide variety of interpersonal contexts, allowing to predict the events in the environment^62^. Regularity and precision of temporal coordination with an external rhythm, were observed to be reduced in healthy older adults^63^. Moreover, Bangert and Balota (2012) compared the continuous tapping accuracy of young and older adults, and individuals in the earliest stages of dementia. They found that young adults outperformed both older adult groups in both measures of accuracy and response variability^64^.

Traditional models of interval timing^65–67^ are composed of three stages: 1) At the onset of a to-be-timed interval, a pacemaker or internal clock encodes time units; 2) These time units are stored in a memory system; 3) When a decision needs to be made about whether a given interval is similar to an earlier perceived interval, these time units are compared, and a decision is made. Following cognitive investigations^68,69^ work on the neural bases of time processing led researchers to several frameworks^70^. According to the striatal beat frequency (SBF)^71^ model, frequently discussed in the neural interpretation of behavioral differences across groups^17^ the start signal to time a given stimulus or event involves oscillatory cortical synchrony. Such phase synchrony would then be detected by the striatum, the central clock in the model, which would then project its activity to the thalamus, the hippocampus, and the cortex^72^. This model thus strongly relies on the precision of synchronized brain couplings^73^. Alternative, distributed models have been proposed, without the presence of a single region detecting coincident events between oscillators as an internal clock^25,74^. These models notably involve state-dependent networks, where temporal information is reflected in the intrinsic and distributed synchronized dynamics of brain connectivity ^70^. However, these models were mainly developed based on findings in animals and in young adults and the evolution of such complex interactions with advancing age remains seldom investigated.

Results also revealed a negative direct effect of aging on Subjective Temporal Distances. We observed that the older people were, the closer they judged the weeks and months ahead. Decoupling from the present and exploring other times^75^, involves multiple cognitive processes, such as episodic memory and the abstraction of temporal relations between events^75,76^, which are also known to decline with advancing age^77^. In summary, the present work specifies that aging mainly affects the aspects tied to order and simultaneity^13^ (on a time scale of the order of 1 s) and the subjective future temporal distance^14,16^, of the next week and month. Conversely, in line with previous systematic questionnaires studies reporting subtle or no changes in the perceived passage of time with age^34,35^, we did not observe a unique effect of age on passage of time judgments concerning the last few days. While a slowing down of the flow of time in presence of feelings like boredom^78^ and sadness^37,79^ has indeed been observed and documented by previous studies, the association with aging effects was not previously been considered.

Here, we observed for the first time that the intensity of depressive symptoms fully mediates the feeling of the passage of time across the lifespan, suggesting these symptoms could play a pivotal role in age-related changes in the subjective experience of the felt passage of time over the last few days. Temporal processing is significantly altered in major depression^80,81^ with reports of a slowing both duration processing and sense of time flow. Dopaminergic deficits and altered nigro-striatal-prefrontal connectivity have been reported in presence of depressive symptoms^82^, and in association with impairments of attention and working memory performance^83^. Changes in the passage of time could thus constitute an indicator of altered cognitive functioning or mental health issues. The present results suggest that aging effects on felt passage of time largely result from the presence of depressive symptoms that may slow down the encoding of time units^84^. More prominently with advancing age, the presence of depressive symptoms could be associated with a lower drift rate or neural trajectory over time. These findings could also reflect a slowing down of the internal clock, and a less precise maintenance and encoding of temporal information with advancing age, associated with decreased memory and attentional capacities^40^. Aging effects on time processing may emerge as the result of even subtle dysfunction of fronto-striatal circuits^85^, which are known to be affected in Major Depression Disorder^86^.

The association between age, depressive symptoms and passage of time is also relevant for frameworks of cognitive aging^3^. For instance, a slower feeling of the passage of time has been showed to distinguish between clinical and sub-clinical depression in the young population^87^. Distortions of temporal aspects of cognition, such as the subjective feeling of the passage of time, could contribute to better identify depressive symptoms in the aging population, and be sensitive to cognitive changes with advancing age. This work extends our knowledge on cognitive changes associated with the presence of depressive symptoms in the aging population and suggest this aspect should be further considered in neuropsychological assessments^13^.

## Future implications

Our results could help to improve the neuropsychological assessment of cognitive changes with advancing age by including easy tasks involving synchronizing with an external rhythm. Resynchronization is important in aging to enhance interpersonal interactions as well as to interact with the environment^63^. Most importantly, the observed slowdown of the passage of time in aging associated with the presence of depressive symptoms could help to detect even sub-clinical depressive symptoms in the aging population, thus clarifying the inter-individual heterogeneity of the trajectories of cognitive decline^3^. However, further longitudinal neuroimaging studies are needed to bridge the gap between the changes of cerebral mechanisms supporting temporal processing in the latest stages of life, and the influence of dopamine neurotransmission changes related to depressive symptoms on temporal processing.

## Limitations

Despite the variety of temporal processing tasks included in the Blursday Project, not every subject enrolled accomplished all the tasks, resulting in different sample sizes for each task. Also, it is worth mentioning that education levels and general cognitive performance measures were not collected in the Blursday Project, which prevented the investigation of the association of the reported results with these variables. Thus, future longitudinal studies will further investigate the variability of aging effects on temporal processing and their associations with other cognitive processes.

## Supplementary Information

Supplementary Information.

## Data Availability

All data are available on the Blursday platform^33^.
