# Supplementary material for: The role of depressive symptoms in the interplay between aging and temporal processing
Source: Sci Rep. 2023 Jul 14;13:11375. doi: 10.1038/s41598-023-38500-3 (PMC10349066; doi:10.1038/s41598-023-38500-3)
Supplement: Supplementary file 1 — Supplementary Information. [file 41598_2023_38500_MOESM1_ESM.pdf]

The role of depressive symptoms in the interplay between aging and temporal processing

Giulia Buzi[1], Francis Eustache[1], Arnaud D'Argembeau[2] and Thomas Hinault[1*]

[1] U1077 INSERM-EPHE-UNICAEN, Caen, 14000, France.
[2] Department of Psychology, Psychology and Neuroscience of Cognition Research Unit, University of Liège, Place des Orateurs 1 (B33), 4000 Liège, Belgium
*Thomas.hinault@inserm.fr

SUPPLEMENTARY MATERIALS

Sample Description

χ^2 Tests for proportions of gender

Age Group		Value	df	p
20-30	χ^2 continuity correction	0.0929	1	0.761
	N	28		
30-40	χ^2 continuity correction	0.7167	1	0.397
	N	27		
40-50	χ^2 continuity correction	0.3775	1	0.539
	N	28		
50-60	χ^2 continuity correction	0.0000	1	1.000
	N	35		
60-80	χ^2 continuity correction	2.4609	1	0.117
	N	30		
Total	χ^2 continuity correction	2.6277	1	0.105
	N	148		

Table S1A

Contingency table showing proportions for gender in each age group.

χ^2 Tests for proportions for region of the world in each age group

REGION		Value	df	p
ASIA	χ^2 continuity correction	3.54	4	0.472
	N	71		
EUROPE	χ^2 continuity correction	7.66	4	0.105
	N	77		
Total	χ^2 continuity correction	7.49	4	0.112
	N	148		

Table S1B
Chi-square test of equality proportions for region of the world

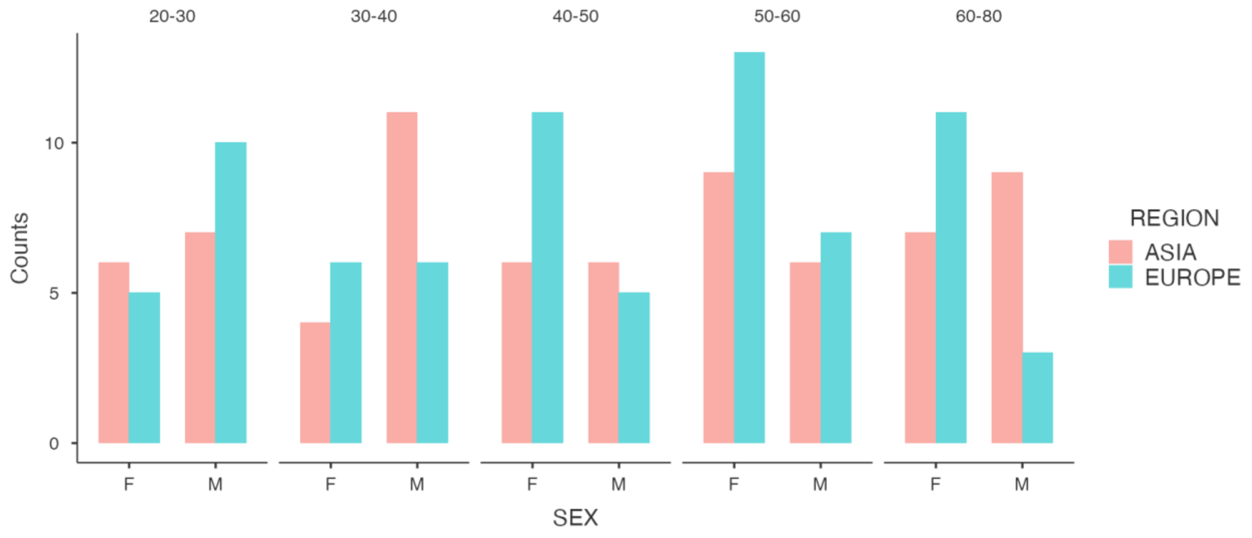

Figure S1.

Histograms showing the sample composition.

Cultural differences

The 5*2 ANOVA to control for cultural differences on HADS-D scores between age groups, highlighted an effect of the region of the world indeed ($F_{(1, 138)}$ = 55.97, p<.001, η²= 0.265), with a higher score for Asian participants than the European ones ($t_{(138)}$ = 7.47, p<.001, d = 1.24). However, a main effect of age ($F_{(4, 138)}$ = 2.503, p = 0.045, η² = 0.047) was reported too, but no significant post-hoc comparisons have been detected by using Bonferroni correction for multiple comparisons. Lastly, no interaction effects were detected ($F_{(4, 138)}$ = 1.368, p = 0.137, η² = 0.034).

Cultural Differences

Cases	Sum of Squares	df	Mean Square	F	p	η²
Region	42.925	1	42.925	55.797	< .001	0.265
Age group	7.702	4	1.926	2.503	0.045	0.047
Region ✻ age group	5.472	4	1.368	1.778	0.137	0.034
Residuals	106.164	138	0.769			

Tables S2A 2*5 ANOVA probing cultural differences in the expression of sadness. *Note.* Type III Sum of Squares. *Note.* Results are averaged over the levels of region.

REGION		REGION	Mean Difference	SE	df	t	$P_{bonferroni}$	Cohen's d	Lower	Upper
Asia	-	Europe	1.09	0.146	138	7.47	< .001***	1.24	0.904	1.58

Table S2B

Post-hoc comparisons on the HADS-D score between region of the world. Multiple comparisons are adjusted by Bonferroni correction.

Post Hoc Comparisons - Age Group

Comparison									95% Confidence Interval	
Age Group		Age Group	Mean Difference	SE	df	t	$p_{bonferroni}$	Cohen's d	Lower	Upper
20-30	-	30-40	-0.36340	0.238	138	-1.5293	1.000	-0.4143	-0.9523	0.124
	-	40-50	0.07431	0.236	138	0.3150	1.000	0.0847	-0.4473	0.617
	-	50-60	0.27419	0.224	138	1.2255	1.000	0.3126	-0.1931	0.818
	-	60-80	0.26443	0.231	138	1.1446	1.000	0.3015	-0.2206	0.824
30-40	-	40-50	0.43771	0.239	138	1.8350	0.687	0.4990	-0.0420	1.040
	-	50-60	0.63759	0.226	138	2.8154	0.056	0.7269	0.2091	1.245
	-	60-80	0.62783	0.234	138	2.6867	0.081	0.7158	0.1822	1.249
40-50	-	50-60	0.19988	0.225	138	0.8896	1.000	0.2279	-0.2794	0.735
	-	60-80	0.19012	0.232	138	0.8196	1.000	0.2168	-0.3068	0.740
50-60	-	60-80	-0.00976	0.220	138	-0.0445	1.000	-0.0111	-0.5060	0.484

Table S2C

Post-hoc comparisons on the HADS-D score between age group. Multiple comparisons are adjusted by Bonferroni correction.

Results

Temporal processing abilities across different age groups

Age group	Psihat	ci.lower	ci.upper	p-value
**20-30 vs. 30-40**	-0.462	-1.328	0.270	0.077
**20-30 vs. 40-50**	0.254	-0.398	0.858	0.274
**20-30 vs. 50-60**	-0.193	-0.930	0.467	0.396
**20-30 vs. 60-80**	-0.763	-2.029	0.480	0.077
**30-40 vs. 40-50**	**0.716**	**0.001**	**1.429**	**0.004**
**30-40 vs. 50-60**	0.268	-0.411	1.032	0.312
**30-40 vs. 60-80**	-0.302	-1.618	0.997	0.515
**40-50 vs. 50-60**	-0.447	-1.001	0.228	0.065
**40-50 vs. 60-80**	**-1.017**	**-2.233**	**0.166**	**0.017**
**50-60 vs. 60-80**	-0.570	-1.961	0.595	0.197

Table S3. Robust post-hoc comparisons between age groups for asynchrony. P-values are adjusted for comparing a family with Bonferroni correction.

Mediation Analyses

Total effect of aging on temporal processing (c path)

The analysis of the total effect of age on temporal processing performance highlighted a positive relationship with asynchrony, suggesting that the ability to maintain the pace after observing a cue, decreases with advancing age ($\beta = 0.129$, $z = 2.113$, $p = 0.035$, 95% CI [-0.021;0.281], Figure 3, panel a). An effect of aging was also observed on the subjective temporal distance of the following week ($\beta = -0.212$, $z = -3.125$, $p = 0.002$, , 95% CI [-0.339;-0.091], Figure 3, panel b) and month ($\beta = - 0.152$, $z = -2.351$, $p = 0.024$, , 95% [-0.272;-0.023], Figure 3, panel c) which were perceived as shorter by older participants.

	n	Estimate	SE	z-value	p	95% Confidence Interval	
						Lower	Upper
Age (z) → Foreperiod Implicit Timing (z)	299	-0.007	0.067	0.106	0.915	-0.128	0.148
Age (z) → Retrospective Duration (z)	175	-0.033	0.090	-0.365	0.715	-0.298	0.147
Age (z) → Spontaneous tapping (z)	321	0.008	0.071	0.112	0.911	-0.051	0.076

**Age** (z) → **Synchronization-Continuation** (z)	364	**0.129**	**0.061**	**2.113**	**0.035***	**-0.021**	**0.281**
Age (z) → Passage of time (z)	306	0.034	0.064	0.521	0.602	-0.094	0.156
**Age** (z) → **Subjective temporal distance (w)** (z)	290	**-0.212**	**0.068**	**-3.125**	**0.002***	**-0.339**	**-0.091**
**Age** (z) → **Subjective temporal distance (m)** (z)	290	**-0.152**	**0.068**	**-2.251**	**0.024***	**-0.272**	**-0.023**

Table S4

Total effects of age (X) on each temporal task (Y). Coefficients are completely standardized, bias-corrected percentile bootstrap confidence intervals, ML estimator. NB: Not all bootstrap samples were successful: CI based on 4999 samples.

* p <0.005.

Path coefficients

						95% Confidence Interval	
		**Estimate**	**Std. Error**	**z-value**	**p**	**Lower**	**Upper**
HADS-D (z)	→ Spontaneous Tapping (z)	-0.076	0.062	-1.222	0.222	-0.161	-0.018
Age (z)	→ Spontaneous Tapping (z)	0.021	0.066	0.319	0.750	-0.032	0.093
HADS-D (z)	→ Synchronization-Continuation (z)	0.016	0.059	0.270	0.787	-0.098	0.131
**Age** (z)	→ **Synchronization-Continuation** (z)	**0.115**	**0.059**	**2.062**	**0.039**	**0.004**	**0.223**
HADS-D (z)	→ Subjective temporal distance (w) (z)	-0.036	0.064	-0.558	0.577	-0.155	0.096
**Age** (z)	→ **Subjective temporal distance (w)** (z)	**-0.206**	**0.068**	**-3.157**	**0.002**	**-0.337**	**-0.081**

Path coefficients

						95% Confidence Interval	
		Estimate	Std. Error	z-value	p	Lower	Upper
HADS-D (z)	→ Passage of time (z)	-0.162	0.061	-2.638	0.008	-0.276	-0.045
Age (z)	→ Passage of time (z)	0.056	0.062	0.901	0.368	-0.064	0.172
HADS-D (z)	→ Subjective temporal distance (m) (z)	-0.081	0.064	-1.259	0.208	-0.202	0.046
**Age** (z)	→ **Subjective temporal distance (m)** (z)	**-0.133**	**0.069**	**-2.033**	**0.042**	**-0.264**	**-0.007**
HADS-D (z)	→ Retrospective Duration (z)	-0.043	0.078	-0.551	0.581	-0.142	0.038
Age (z)	→ Retrospective Duration (z)	-0.017	0.084	-0.201	0.841	-0.261	0.155
HADS-D (z)	→ Foreperiod Implicit Timing (z)	-0.114	0.066	-1.721	0.085	-0.242	0.008
Age (z)	→ Foreperiod Implicit Timing (z)	0.019	0.064	0.305	0.761	-0.107	0.158
**Age** (z)	→ **HADS-D** (z)	**0.176**	**0.058**	**3.060**	**0.002**	**0.070**	**0.286**
**HADS-A** (z)	→ **Age** (z)	**-0.280**	**0.054**	**-5.228**	**< .001**	**-0.392**	**-0.169**
Confinement Index (z)	→ Age (z)	-0.026	0.053	-0.486	0.627	-0.122	0.083
**HADS-A** (z)	→ **HADS-D** (z)	**0.333**	**0.055**	**6.075**	**< .001**	**0.223**	**0.446**
Confinement Index (z)	→ HADS-D (z)	-0.021	0.052	-0.393	0.694	-0.134	0.085
HADS-A (z)	→ Spontaneous Tapping (z)	0.109	0.068	1.588	0.112	0.004	0.273
Confinement Index (z)	→ Spontaneous Tapping (z)	0.047	0.060	0.780	0.436	-0.030	0.179
HADS-A (z)	→ Synchronization-Continuation (z)	-0.002	0.061	-0.032	0.975	-0.124	0.125
Confinement Index (z)	→ Synchronization-Continuation (z)	-0.051	0.056	-0.905	0.365	-0.144	0.047
HADS-A (z)	→ Subjective temporal distance (w) (z)	-0.070	0.070	-1.013	0.311	-0.210	0.074
Confinement Index (z)	→ Subjective temporal distance (w) (z)	0.028	0.061	0.450	0.653	-0.088	0.147
HADS-A (z)	→ Passage of time (z)	0.010	0.064	0.151	0.880	-0.100	0.131
Confinement Index (z)	→ Passage of time (z)	-0.063	0.061	-1.035	0.301	-0.189	0.060
HADS-A (z)	→ Subjective temporal distance (m) (z)	0.014	0.070	0.206	0.836	-0.124	0.166

Path coefficients

							95% Confidence Interval	
			Estimate	Std. Error	z-value	p	Lower	Upper
Confinement Index (z)	→	Subjective temporal distance (m) (z)	-0.073	0.062	-1.181	0.238	-0.208	0.048
HADS-A (z)	→	Retrospective Duration (z)	-0.115	0.092	-1.255	0.209	-0.367	0.046
Confinement Index (z)	→	Retrospective Duration (z)	-0.105	0.080	-1.308	0.191	-0.336	0.025
HADS-A (z)	→	Foreperiod Implicit Timing (z)	0.064	0.067	0.959	0.338	-0.084	0.217
Confinement Index (z)	→	Foreperiod Implicit Timing (z)	-0.011	0.061	-0.174	0.862	-0.136	0.125

Table S5. Path coefficient estimates. Delta method standard errors, bias-corrected percentile bootstrap confidence intervals, ML estimator. NB: Not all bootstrap samples were successful: CI based on 4999 samples.

Mediation Analyses on the balanced subsample (n=148)

Direct effects

								95% Confidence Interval	
			n	Estimate	Std. Error	z-value	p	Lower	Upper
Age	→	Passage Of Time	135	0.003	0.0005	4.833	< .001	-0.010	0.015

Table S6A

Direct effects of age (X) on Passage Of Time (Y) of the gender and culture balanced subsample (n=148). Coefficients are completely standardized, bias-corrected percentile bootstrap confidence intervals. Not all bootstrap samples were successful: CI based on 4999 samples.

Indirect effects

								95% Confidence Interval	
			**n**	**Estimate**	**Std. Error**	**z-value**	**p**	**Lower**	**Upper**
Age	→	Passage Of Time	135	0.002	0.0002	7.434	< .001	-0.0001	0.005

Table S6B

Indirect effects of Age (X) on Passage Of Time (Y), through the mediation of the HADS-D score, of the gender and culture balanced subsample (n=148). Coefficients are completely standardized, bias-corrected percentile bootstrap confidence intervals. Not all bootstrap samples were successful: CI based on 4999 samples.

Total effects

								95% Confidence Interval	
			**n**	**Estimate**	**Std. Error**	**z-value**	**p**	**Lower**	**Upper**
Age	→	Passage Of Time	135	0.004	0.0005	8.638	< .001	-0.007	0.015

Table S6C

Total effects of age (X) on Passage Of Time (Y) of the gender and culture balanced subsample (n=148). Coefficients are completely standardized, bias-corrected percentile bootstrap confidence intervals. Not all bootstrap samples were successful: CI based on 4999 samples.

Path coefficients

							95% Confidence Interval	
			Estimate	Std. Error	z-value	p	Lower	Upper
HADS-D	→	Passage of time	-0.038	0.0005	-7.459	< .001	-0.078	0.002
Age	→	Passage of time	0.003	0.0005	4.833	< .001	-0.010	0.015
Age	→	HADS-D	-0.047	0.0005	-88.221	< .001	-0.099	0.007
HADS-A	→	Age	-0.559	0.0005	-105.365	< .001	-1.194	0.075
Confinement index	→	Age	-0.124	0.0002	-437.318	< .001	-0.255	0.007
HADS-A	→	HADS-D	0.246	0.0005	50.161	< .001	0.094	0.394
Confinement index	→	HADS-D	0.043	0.0002	156.829	< .001	0.005	0.077

Path coefficients

						95% Confidence Interval	
		Estimate	Std. Error	z-value	p	Lower	Upper
HADS-A	→ Passage of time	-0.006	0.0005	-1.106	0.269	-0.049	0.036
Confinement index	→ Passage of time	-0.001	0.0003	-3.592	< .001	-0.010	0.008

Table S6D

Path coefficients of the General Linear Model of Mediation.
